# Structural Basis for Recruitment of DAPK1 to the KLHL20 E3 Ligase

**DOI:** 10.1016/j.str.2019.06.005

**Published:** 2019-09-03

**Authors:** Zhuoyao Chen, Sarah Picaud, Panagis Filippakopoulos, Vincenzo D'Angiolella, Alex N. Bullock

**Affiliations:** 1 Structural Genomics Consortium, University of Oxford, Old Road Campus, Roosevelt Drive, Oxford OX3 7DQ, UK; 2 Department of Oncology, Cancer Research UK and Medical Research Council Institute for Radiation Oncology, University of Oxford, Oxford OX3 7DQ, UK

**Keywords:** BTB, ubiquitination, Cullin-RING ligase, crystallography, CUL3, protein-protein interaction, cancer, autophagy

## Abstract

BTB-Kelch proteins form the largest subfamily of Cullin-RING E3 ligases, yet their substrate complexes are mapped and structurally characterized only for KEAP1 and KLHL3. KLHL20 is a related CUL3-dependent ubiquitin ligase linked to autophagy, cancer, and Alzheimer's disease that promotes the ubiquitination and degradation of substrates including DAPK1, PML, and ULK1. We identified an “LPDLV”-containing motif in the DAPK1 death domain that determines its recruitment and degradation by KLHL20. A 1.1-Å crystal structure of a KLHL20 Kelch domain-DAPK1 peptide complex reveals DAPK1 binding as a loose helical turn that inserts deeply into the central pocket of the Kelch domain to contact all six blades of the β propeller. Here, KLHL20 forms salt-bridge and hydrophobic interactions including tryptophan and cysteine residues ideally positioned for covalent inhibitor development. The structure highlights the diverse binding modes of β-propeller domains versus linear grooves and suggests a new target for structure-based drug design.

## Highlights

• An “LPDLV” motif in DAPK1 determines its recruitment and degradation by KLHL20
• 1.1-Å crystal structure determined of a KLHL20 Kelch domain-DAPK1 peptide complex
• A DAPK1 helical turn inserts into the β propeller to contact all six Kelch repeats
• KLHL20 shows a hydrophobic binding pocket suitable for inhibitor development

## Graphical Abstract

**Figure:**
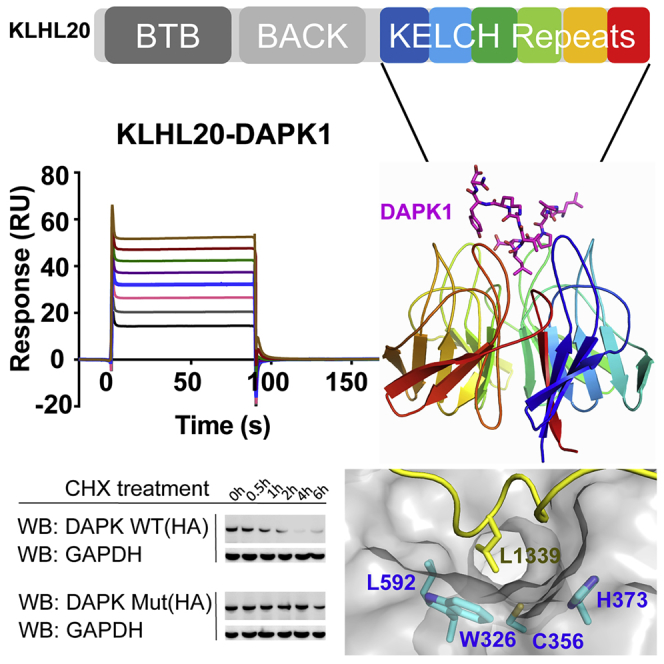

## Teaser

KLHL20 is a BTB-Kelch family E3 ligase linked to autophagy, cancer, and Alzheimer's disease. Chen et al. identify an “LPDLV” motif in DAPK1 that determines its recruitment and degradation by KLHL20. A 1.1-Å crystal structure of a KLHL20-DAPK1 complex reveals a hydrophobic pocket in KLHL20 suitable for inhibitor development.

## Introduction

Ubiquitination by E1-E2-E3 enzyme cascades is the major mechanism by which cells mark proteins for degradation, but can also facilitate protein trafficking, transcriptional control, and cell signaling (Hershko and Ciechanover, 1998, Rape, 2018). The substrate specificity of protein ubiquitination is determined at the level of E3 ubiquitin ligases, which recruit their cognate substrate proteins to complete the enzyme cascade (Buetow and Huang, 2016). Kelch-like protein 20 (KLHL20, also known as KLEIP) is a member of the BTB-Kelch family that assembles with CUL3 and RBX1 to form a multi-subunit Cullin-RING E3 ligase (Geyer et al., 2003, Hara et al., 2004, Pintard et al., 2004). These complex E3 ligases use the RBX1 subunit to engage a charged E2-ubiquitin pair before transferring the ubiquitin to substrates captured by the BTB-Kelch protein (Genschik et al., 2013, Petroski and Deshaies, 2005). Catalysis is enhanced by CUL3 neddylation, which stabilizes the correct geometry of the complex for ubiquitin transfer (Duda et al., 2008).

Like other BTB-Kelch family members, KLHL20 utilizes multiple functional domains. The BTB and 3-box domains confer binding to CUL3, whereas the Kelch β-propeller domain serves as the substrate recognition domain (Canning et al., 2013, Lee et al., 2010). To date, the majority of substrates identified for KLHL20 have been targeted for proteasomal degradation, suggesting their modification by Lys48-linked polyubiquitin chains (Chen et al., 2016). These include the substrates DAPK1 (Lee et al., 2010), PML (Yuan et al., 2011), PDZ-RhoGEF (Lin et al., 2011), and ULK1 (Liu et al., 2016). However, KLHL20 also plays an important role in protein trafficking by targeting coronin 7 to the *trans*-Golgi network through atypical K33-linked polyubiquitination (Yuan et al., 2014).

The substrates of KLHL20 reflect its function in cellular stress responses, as well as its linkage to human disease (Chen et al., 2016). Transcription of the *KLHL20* gene is upregulated by the hypoxia-inducible factor HIF-1α, leading to its overexpression in hypoxic tumor cells (Yuan et al., 2011). In this context, KLHL20 can promote tumorigenesis by degrading the tumor-suppressor proteins DAPK1 and PML. In human prostate cancer patients, higher levels of KLHL20 (and low PML) were found to correlate specifically with high-grade tumors (Yuan et al., 2011). Moreover, KLHL20 depletion in PC3 prostate cancer cells restricted the growth of tumor xenografts, suggesting KLHL20 as a potential therapeutic target (Yuan et al., 2011). KLHL20 also plays a critical role in autophagy termination by degrading the pool of activated ULK1 (Liu et al., 2016). Thus, KLHL20 can restrict both apoptotic and autophagic cancer cell death. Importantly, interferon stimulation causes the sequestration of KLHL20 in so-called PML nuclear bodies, which occur as punctate membraneless substructures of the nucleus enriched with PML protein (Lee et al., 2010). This inhibitory mechanism allows DAPK1 to evade degradation and to accumulate to mediate interferon-induced cell death (Lee et al., 2010). Notably, the stress responses of KLHL20 also appear linked to neurodegeneration, with KLHL20 RNA transcript levels being among the top 20 biomarkers for Alzheimer's disease progression (Arefin et al., 2012, Gomez Ravetti et al., 2010).

Despite the growing number of substrate proteins identified for the 50 members of the BTB-Kelch family, there remains limited knowledge of their specific binding epitopes and consequently a lack of structural information about the corresponding E3-substrate complexes. Here, we investigated the binding of KLHL20 to DAPK1, which was the first reported substrate for this E3 ligase (Lee et al., 2010). Yeast two-hybrid studies previously mapped the interaction to the death domain of DAPK1 and the Kelch domain of KLHL20. It was further shown that the death domain was required for DAPK1 ubiquitination and degradation by KLHL20 (Lee et al., 2010). Through a peptide scanning approach we identified an “LPDLV”-containing recruitment site within this DAPK1 region that bound to KLHL20 with low micromolar affinity. We also determined the crystal structure of their complex at 1.1-Å resolution, revealing a distinct peptide binding mode compared with the previously determined structural complexes of KEAP1 and KLHL3 (Lo et al., 2006, Padmanabhan et al., 2006, Schumacher et al., 2014). The structure further identifies a hydrophobic substrate pocket that appears attractive for small-molecule inhibitor development.

## Results

### Mapping of the DAPK1 Binding Motif for KLHL20 Recruitment

**Figure 1:**
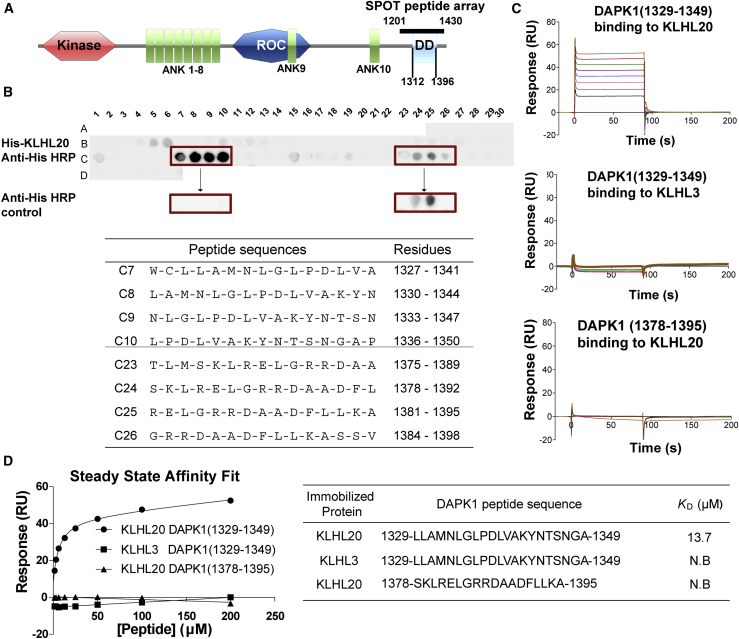
Mapping of the DAPK1 Binding Motif for KLHL20 Recruitment (A) Domain organization of human DAPK1 (ank, ankyrin repeat; DD, death domain comprising residues 1,312–1,396). Solid bar denotes the extended region explored for KLHL20 interaction (DAPK1 residues 1,201–1,430). (B) SPOT peptide array. Each spot was printed as a 15-mer DAPK1 peptide with a 3-residue frameshift at each consecutive position. Arrays were incubated with purified 6xHis-KLHL20 Kelch domain and washed, then KLHL20 binding was detected using anti-His HRP-conjugated antibody. Binding was observed at two sites spanning DAPK1 residues 1,327–1,350 and 1,378–1,395, respectively. As a control, duplicate spots were probed with antibody alone and revealed non-specific antibody binding to DAPK1 residues 1,378–1,395. (C) For SPR experiments, KLHL20 and KLHL3 Kelch domains were immobilized by amine coupling on different flow cells of a CM5 sensor chip. Indicated DAPK1 peptides were injected subsequently at concentrations of 1.6 μM, 3.1 μM, 6.2 μM, 12.5 μM, 25 μM, 50 μM, 100 μM, and 200 μM. Binding was monitored at a flow rate of 30 μL/min. (D) SPR binding data shown are representative of two independent experiments. Data were fitted using a steady-state affinity equation. DAPK1 residues 1,329–1,349 bound to KLHL20 Kelch domain with *K*_D_ = 13.7 μM (N.B., no binding detected).

The recombinant death domain of DAPK1 (Figure 1A) has been shown to display intrinsic disorder and a high propensity for aggregation, making it unsuitable for structural studies (Dioletis et al., 2013). Given the lack of structural order, we set out to map the DAPK1 binding epitope using the SPOT peptide technology. We synthesized a peptide array to span the length of the DAPK1 death domain using 15-mer peptides and a 3-amino-acid frameshift at each position. Probing of the array with recombinant 6xHis-KLHL20 Kelch domain and anti-His-antibody for detection revealed protein capture at two sites encompassing DAPK1 residues 1,327–1,350 and 1,378–1,395, respectively (Figure 1B). A control experiment indicated that the binding epitope was likely to reside within the N-terminal region since peptides from the second site also bound to the anti-His antibody alone, marking them as likely false positives (Figure 1B).

To validate these putative interaction sites, we designed peptides for the two DAPK1 regions and performed surface plasmon resonance (SPR) experiments to measure their respective binding affinities for KLHL20 (Figure 1C). A DAPK1 peptide spanning the N-terminal site residues 1,329–1,349 bound robustly to the Kelch domain of KLHL20 with *K*_D_ = 13.7 μM (Figure 1D). The same peptide showed no apparent binding to the Kelch domain of KLHL3, demonstrating that the interaction was specific to KLHL20 (Figure 1D). A DAPK1 peptide spanning the C-terminal site residues 1,378–1,395 also failed to bind to KLHL20, confirming that this downstream region was a false positive (Figure 1D). Together these data identified a single epitope within the death domain of DAPK1 that showed both potency and specificity for interaction with KLHL20.

### An “LPDLV” Motif in DAPK1 Is Critical for KLHL20 Interaction

**Figure 2:**
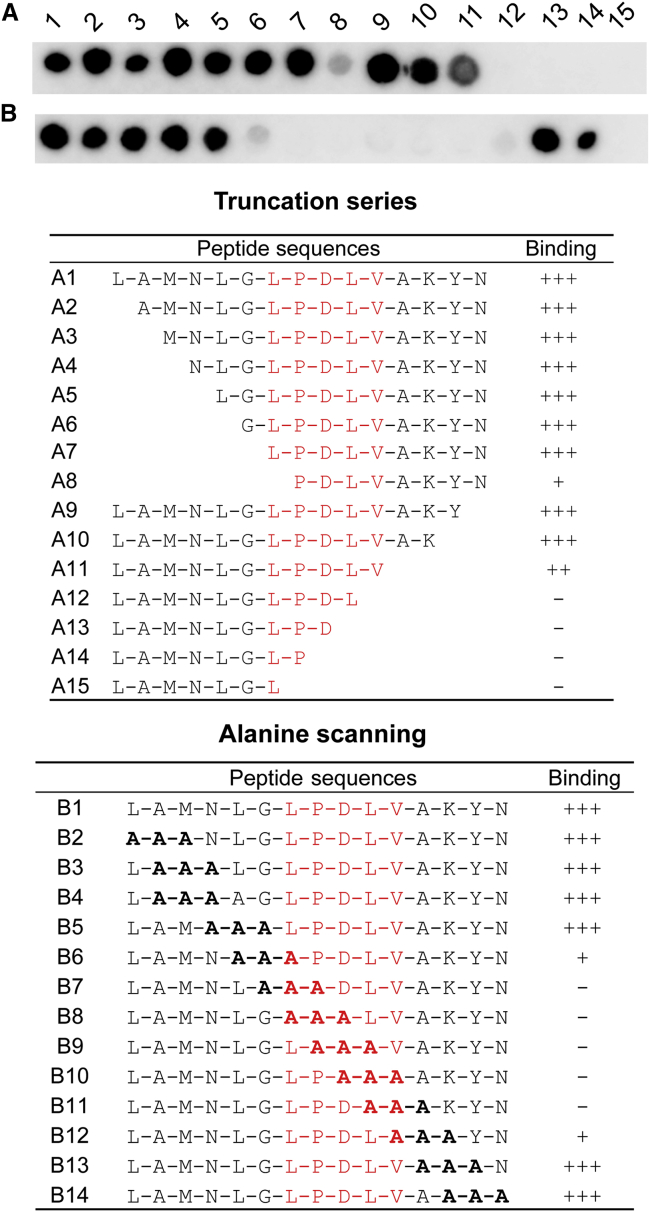
An “LPDLV” Motif in DAPK1 Is Critical for KLHL20 Interaction DAPK1 peptide variants were printed in SPOT peptide arrays. Row-A peptides explored N- and C-terminal truncations, whereas row B explored triple-alanine scanning mutagenesis. Arrays were incubated with purified 6xHis-KLHL20 Kelch domain and washed, then binding was detected with anti-His antibody. KLHL20 binding was abrogated upon deletion or mutation of a central “LPDLV” sequence motif in DAPK1.

Attempts to crystallize KLHL20 either alone or in complex with the identified 21-mer peptide from DAPK1 produced only microcrystalline material yielding poor diffraction. Therefore, we sought to refine the minimal DAPK1 epitope by using the SPOT technology for peptide truncation experiments, as well as alanine scanning to probe the sequence determinants of binding. The results from these experiments were in excellent agreement and identified DAPK1 residues Leu1336 to Val1340 as critical for KLHL20 interaction (Figure 2). N-terminal deletion or mutation of Leu1336 drastically reduced KLHL20 binding, whereas C-terminal deletion or mutation of Val1340 abolished all detectable binding (Figure 2). Other deletions and mutations outside of this region appeared well tolerated, mapping the critical binding region to a “1336-LPDLV-1340” motif in DAPK1.

### High-Resolution Structure of the KLHL20 Kelch Domain Bound to DAPK1 Peptide

**Figure 3:**
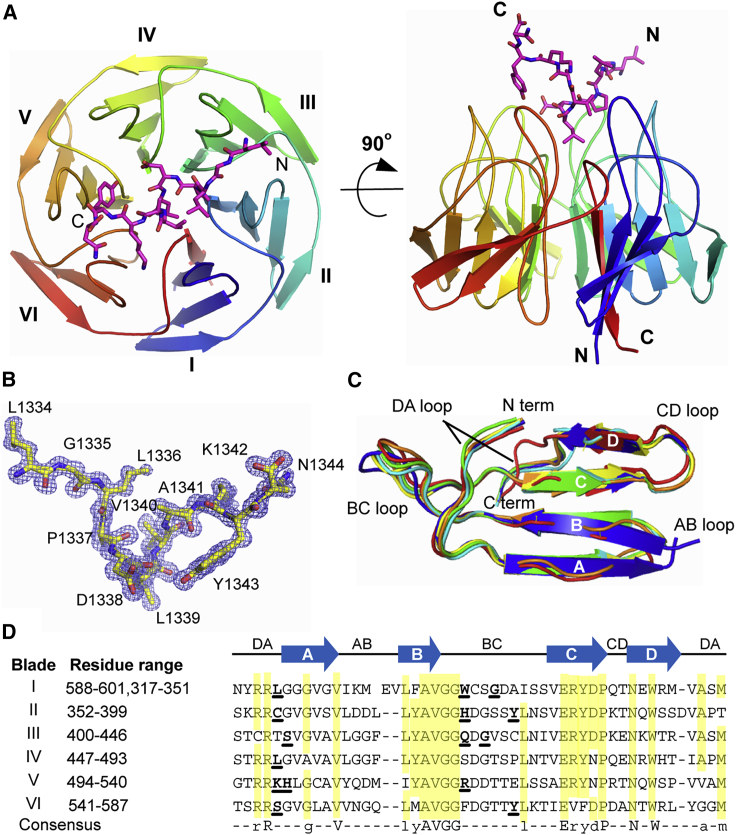
High-Resolution Structure of KLHL20 Kelch Domain Bound to DAPK1 Peptide (A) Overview of the structure of KLHL20 Kelch domain in complex with DAPK1 peptide (purple sticks). Kelch repeats forming blades I to VI are labeled. (B) 2F_o_-F_c_ electron density map (blue mesh) for the DAPK1 peptide contoured at 1.0σ. (C) Superposition of Kelch domain blades I to VI colored from blue to red. Each blade is composed of four antiparallel β strands (labeled A–D) and connecting loops. (D) Sequence alignment of the six Kelch repeats in KLHL20. Conserved residues are highlighted in yellow. DAPK1-interacting residues are shown in bold and underlined.

For further co-crystallization trials, we tried an 11-residue DAPK1 peptide (LGLPDLVAKYN) in order to capture interactions of the central “LPDLV” motif, while allowing for local conformational preferences and potential flanking interactions. Viable crystals were obtained with strong diffraction after a combination of microseeding from initial hits and fine matrix screening for further optimization. Subsequently, we were able to determine a high-resolution structure for the complex of KLHL20 and DAPK1 peptide (Table 1). The structure was refined at 1.1-Å resolution and traced the full KLHL20 Kelch domain from residues 317 to 601 (Figure 3A). The complete DAPK1 peptide was also clearly defined in the electron density map (Figure 3B), allowing its binding interactions to be mapped in atomic detail.

**Table 1:** Data Collection and Refinement Statistics for the KLHL20 Kelch Domain-DAPK1 Complex

**Data Collection**	

Wavelength (Å)	0.9763
Space group	*P*2_1_2_1_2_1_
Unit cell dimensions	
*a*, *b*, *c* (Å)	40.5, 47.4, 151.9
α, β, γ (°)	90, 90, 90
Total reflections	242,242 (19,836)
Unique reflections	122,957 (11,329)
Multiplicity	2.0 (1.8)
Completeness (%)	99 (92)
*I*/σ	12.89 (2.75)
*R*_meas_	0.0424 (0.293)
CC_1/2_	0.998 (0.902)

**Refinement**	

Resolution range (Å)	40.2–1.09 (1.13–1.09)
*R*_work_	0.154 (0.221)
*R*_free_	0.173 (0.216)
RMSD bonds (Å)	0.01
RMSD angles (°)	1.32
Wilson *B* factor (Å^2^)	9.65
Ramachandran favored (%)	97.8
Ramachandran allowed (%)	2.2

Values in parentheses indicate highest-resolution shell. RMSD, root-mean-square deviation.

The Kelch domain structure shows a canonical β-propeller fold. The six Kelch repeats form the six blades (I to VI) of the propeller arranged radially around a central axis. Each repeat is folded into a twisted β sheet consisting of four antiparallel β strands (A to D, Figure 3C). A final C-terminal β strand is observed to close the β propeller and inserts into blade I as the innermost βA strand. Blade I is therefore composed of a C-terminal βA strand and N-terminal βB, βC, and βD strands. Packing between each blade is mediated by a number of conserved hydrophobic positions as well as several buried charged residues that recur within each Kelch repeat (Figure 3D).

The substrate binding surface on KLHL20 is shaped by the long BC loops, which protrude outward from the Kelch domain surface, and the largely buried DA loops, which link adjacent blades and contribute to the protein core. Notably, the six BC loops in KLHL20 are all of equal length comprising 11 residues (Figure 3D), whereas other Kelch domain structures have shown more varied loop lengths across the different blades (Canning et al., 2013).

### Extended Interactions of the DAPK1 Peptide

**Figure 4:**
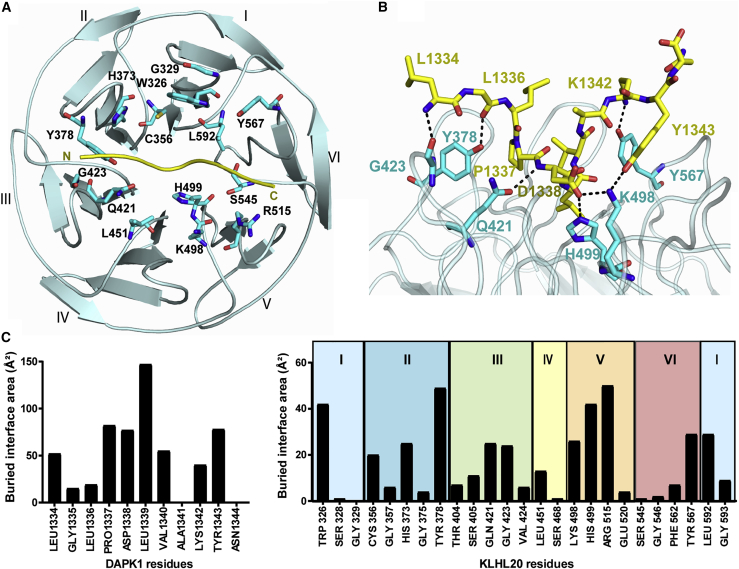
Interactions in the KLHL20 Kelch Domain-DAPK1 Complex (A) An overview of the DAPK1 binding residues in KLHL20 colored cyan with DAPK1 peptide shown as a yellow ribbon. (B) Salt-bridge and hydrogen-bond interactions in the complex interface are shown by dashed lines. (C) Buried interface surface areas for interacting residues in the KLHL20 Kelch domain-DAPK1 complex. See also Figure S1 for water-mediated interactions.

The bound DAPK1 peptide shows an extended conformation that packs between Kelch domain blades II and III at its N terminus and blades V and VI at its C terminus (Figure 4A). At its center, the peptide adopts a single loose helical turn that is stabilized by intramolecular hydrogen bonds between the carbonyl of Pro1337 and the amides of Val1340 and Ala1341. Here, the peptide inserts deeply into the central cavity of the Kelch domain β propeller where it is anchored in the complex by Leu1339, the second leucine in the “LPDLV” motif (Figure 4B). Binding at this central region allows the peptide to form additional contacts with blades I and IV. Thus, DAPK1 forms interactions with all six Kelch repeats, including interactions with all six DA loops and all BC loops with the exception of the BC loop in blade IV (Figures 3D and 4C).

### Interactions of the “LPDLV” Motif

**Figure 5:**
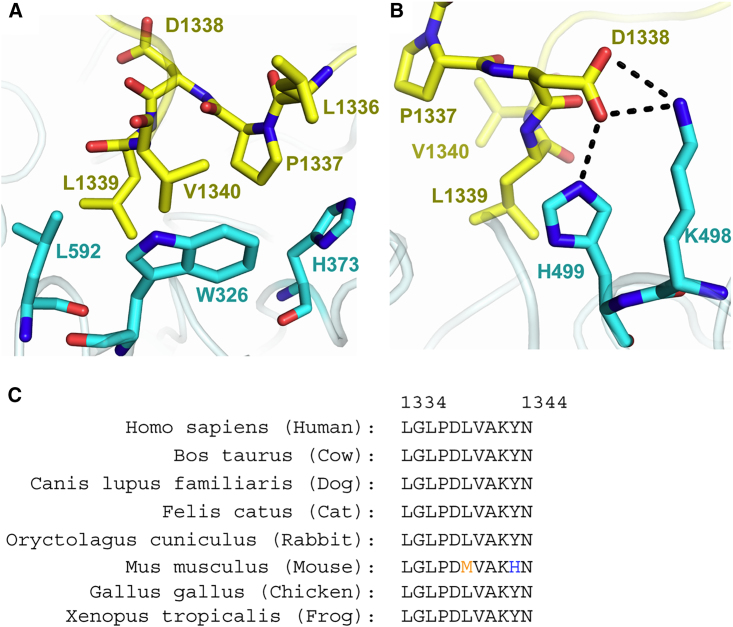
Interactions of the DAPK1 “LPDLV” Motif (A) Hydrophobic interactions between the DAPK1 “LPDLV” motif and KLHL20 Kelch domain blades I and II. (B) DAPK1 Asp1338 forms a salt bridge with KLHL20 Lys498, as well as a hydrogen bond to H499. (C) The sequence of the crystallized DAPK1 peptide is conserved across species.

The “LPDLV” motif of DAPK lies at the core of the protein-peptide interface. Here, the hydrophobic side chains pack against Kelch domain blades I and II and make notable van der Waals contacts with KLHL20 Trp326, His373, and Leu592, respectively (Figure 5A). Somewhat surprisingly, the first leucine in the “LPDLV” motif, Leu1336, is oriented away from the binding interface and has only minor interaction with KLHL20, mostly through its main-chain atoms. In the SPOT peptide arrays, changes at this position reduced KLHL20 binding significantly but did not abolish it (Figure 2). The importance of this position likely stems from the conformational constraints of the following DAPK1 residue, Pro1337. By contrast, the second leucine, Leu1339, is the most buried DAPK1 residue in the complex (Figure 4C). This side chain lies sandwiched between KLHL20 His499 and Leu592 (Figures 5A and 5B), but forms interactions across all the Kelch repeats, except for blade IV, by virtue of its central binding position. The final residue in the “LPDLV” motif, DAPK1 Asp1338, is oriented away from the hydrophobic side chains to face Kelch domain blade V, where it forms a salt bridge with KLHL20 Lys498 as well as a hydrogen bond to His499 (Figure 5B). Residues across the mapped DAPK1 binding motif are well conserved across vertebrate species (Figure 5C).

### Direct and Water-Mediated Hydrogen Bonding in the KLHL20 Kelch Domain-DAPK1 Complex

In total, the complex between KLHL20 and DAPK1 includes eight direct hydrogen-bond or salt-bridge interactions (Figure 4B), as well as a number of water-mediated interactions (Figure S1). The N-terminal three residues of the DAPK1 peptide are oriented away from the KLHL20 surface. Their binding interactions are mediated by their main-chain atoms, which form hydrogen bonds with KLHL20 residues Tyr378, Gln421, and Gly423, respectively (Figure 4B). Gln421 forms an additional hydrogen bond with the backbone amide of DAPK1 Asp1338, one of the critical residues within the “LPDLV” motif. Toward the C terminus of the peptide, interactions are formed through DAPK1 Lys1342 and Tyr1343, while Ala1341 and Asn1344 are oriented to solvent. Lys1342 folds toward Kelch domain blade VI where it forms a direct hydrogen bond to the BC loop residue Tyr567 (Figure 4B). DAPK1 Tyr1343 folds instead against blade V to hydrogen bond with KLHL20 Lys498 (DA loop, Figure 4B) and forms additional hydrophobic packing with the BC loop residue Arg515. Water molecules in the complex help to bridge more distant contacts or to satisfy other nitrogen and oxygen atoms that otherwise lack direct hydrogen bonds (Figure S1).

### KLHL20-Induced Degradation of DAPK1 Is Dependent on the “LPDLV” Motif

**Figure 6:**
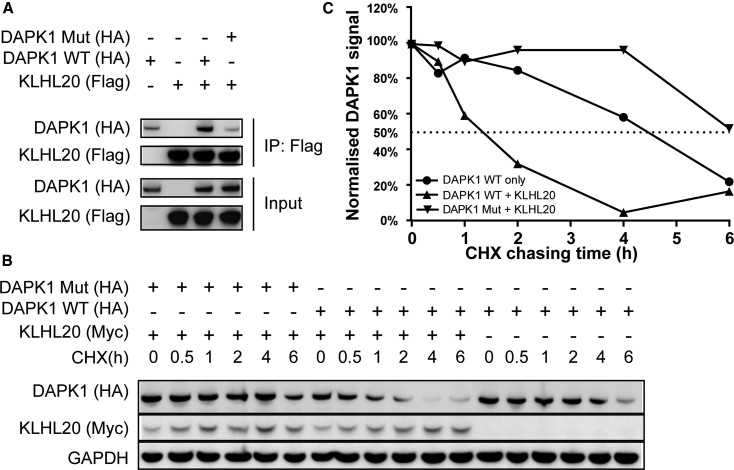
Mutations in the DAPK1 “LPDLV” Motif Impair DAPK1 Binding and Degradation by KLHL20 (A) Full-length DAPK1 variants and KLHL20 Kelch domain were co-transfected into HEK293T cells as indicated. FLAG-KLHL20 Kelch domain was immunoprecipitated (IP) with anti-FLAG antibody. DAPK1 WT was robustly co-purified with KLHL20, whereas DAPK1 mutant was only recovered at the background level of the beads alone. (B) DAPK1 variants were transfected into HEK293T cells with or without full-length KLHL20 as indicated. At 24 h post transfection, cells were incubated with 100 μg/mL cycloheximide (CHX) and harvested at different time points as indicated. DAPK1 protein levels were detected by western blot and normalized to GAPDH. (C) Quantitation of (B).

To confirm the identified “LPDLV” motif as a regulatory site for DAPK1 interaction and degradation, we performed immunoprecipitation and cycloheximide (CHX) chase experiments in HEK293T cells. To disrupt the interaction site, we generated a full-length DAPK1 mutant in which the critical 1,336-“LPDLV” motif was mutated to “LPAAV.” Immunoprecipitation (Figure 6A) of FLAG-KLHL20 Kelch domain and HA-DAPK1 full-length variants showed that the wild-type (WT) DAPK1 was robustly bound to KLHL20 whereas the DAPK1 mutant was only recovered at a low background level also observed with anti-FLAG agarose beads alone. We then performed a CHX chase assay to compare the degradation of DAPK1 variants in the presence or absence of full-length KLHL20. As shown in Figures 6B and 6C, co-expression of full-length KLHL20 and DAPK1 WT caused a striking reduction in the half-life of DAPK1 compared with expressing DAPK1 WT alone. However, the DAPK1 mutant appeared strikingly resistant to KLHL20 co-expression, consistent with its disrupted protein interaction. Indeed, the half-life of the DAPK1 mutant exceeded that of DAPK1 WT under any of the conditions above. Taken together, these data indicated that the “LPDLV” motif was required for both DAPK1 recruitment and degradation by KLHL20.

## Discussion

The Cullin-RING E3 ligase KLHL20 has been shown to ubiquitinate some half a dozen protein targets that link its activities to diverse processes including autophagy, hypoxia, cancer, and Alzheimer's disease (Chen et al., 2016). Here, we performed the first structural and biochemical analyses of KLHL20 to elucidate how it engages its substrates through the Kelch β-propeller domain. Structural studies required the identification of a short DAPK1 peptide motif that subsequently enabled crystallization. As a result, we were able to solve the structure of the KLHL20 Kelch domain in complex with DAPK1 peptide at 1.1-Å resolution. The structure identifies a central “LPDLV” motif in the DAPK1 epitope that inserts into the central pocket of the Kelch β propeller as a loose helical turn. The interface in KLHL20 complements this motif with a hydrophobic core supported by a salt-bridge interaction. The recognition motifs within other KLHL20 substrates remain to be defined at the same level, but are likely to form a similar pattern of hydrophobic and charge-charge interactions. Indeed, EPAS1 is another reported interaction partner of KLHL20 (Higashimura et al., 2011) and contains a 691-“GPDVL” motif in its C-terminal region for which we could also detect binding to the KLHL20 Kelch domain in a SPOT array (Figure S2).

**Figure 7:**
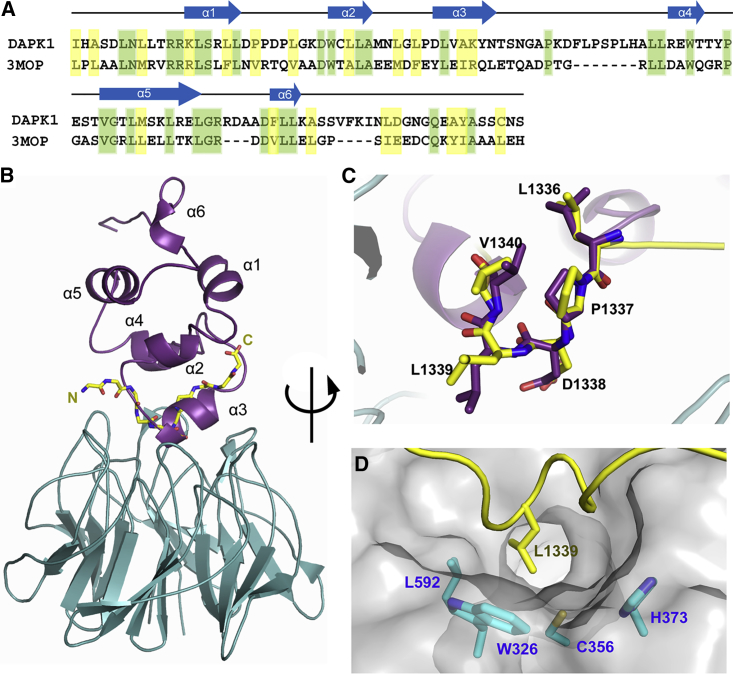
Homology Model of DAPK1 Death Domain in Complex with KLHL20 Kelch Domain (A) Sequence alignment of the death domains of DAPK1 and MyD88 (PDB: 3MOP, chain A). (B) Superposition of the KLHL20 Kelch domain-DAPK1 structure (cyan/yellow) and a homology model of the DAPK1 death domain (purple; template PDB: 3MOP) based on the critical “LPDLV” motifs. A similar comparison with the template structure is shown in Figure S3. (C) Close-up view showing good agreement between the helical conformation of the “LPDLV” motif in the crystallized DAPK1 peptide and the homology model (α3). (D) Surface representation of KLHL20 highlighting the potentially druggable pocket bound by DAPK1 Leu1339. In addition to hydrophobics such as KLHL20 Trp326, the proximal location of KLHL20 Cys356 suggests opportunity for the development of covalent inhibitors.

The low micromolar binding of the DAPK1 peptide to KLHL20 is comparable in affinity with substrates of the SPOP E3 ligase (Zhuang et al., 2009), which similarly assembles into a Cullin-RING ligase complex through CUL3 (Errington et al., 2012). However, this is weaker than the low nanomolar binding observed for NRF2 interaction with the Kelch domain of KEAP1 (Tong et al., 2006). These differences may reflect the strict regulation of NRF2, which ensures its constitutive degradation, or the requirement for substrate co-adaptors as found for KLHL12 (McGourty et al., 2016). Alternatively, there may be differences in affinity between the binding of DAPK1 peptide and the death domain in the context of the full-length DAPK1 or KLHL20 proteins. Death domains are well-known protein interaction modules that fold as a bundle of six α helices (Ferrao and Wu, 2012). While the isolated death domain of DAPK1 appears to be intrinsically disordered, it is possible that other DAPK regions contribute to its proper folding (Dioletis et al., 2013). The critical “LPDLV” motif of DAPK1 maps to the predicted α3 helix. To understand how this might interact with KLHL20 in the context of the full death domain, we built a homology model of human DAPK1 using the MyD88 protein structure (PDB: 3MOP) as a template and ICM-Pro software (Molsoft) (Figures 7A and 7B) (Abagyan et al., 1997). Superposition of the “LPDLV” motifs revealed good agreement between the model, the MyD88 template, and our crystal structure (Figures 7B, 7C, and S3). Overall, the helical turn of the DAPK1 peptide was a good match to the folding of the α3 helix (Figures 7C and 7D). Consequently, residues in the “LPDLV” motif were closely aligned in the different structures (Figure 7C). However, structural deviations in the flanking peptide residues suggest that their interactions are less certain in the context of the folded death domain. Most importantly, the key interacting residues of DAPK1 were exposed, providing a surface epitope for KLHL20 to bind. The model suggests that the α3 helix of the death domain can insert into the relatively wide pocket of KLHL20 to recapitulate the observed peptide interaction without steric hindrance. There is some precedent for such an arrangement from the structure of KEAP1 bound to the DLG motif of NRF2, which also formed an extended helical structure (Fukutomi et al., 2014).

Of note, previous studies of KEAP1 have characterized the binding of both unmodified and phosphorylated peptides (for example, the “ETGE”-containing motif from NRF2 and the “phospho-STGE”-containing motif of sequestosome-1/p62) (Ichimura et al., 2013, Lo et al., 2006, Padmanabhan et al., 2006). To date, no post-translational modifications have been reported for the death domain of DAPK1 (www.phosphosite.org [Hornbeck et al., 2015]), and we have yet to identify a phosphorylated substrate motif for KLHL20. Nonetheless, other substrates of KLHL20 may similarly substitute a phosphorylated residue for the aspartate found in the “LPDLV” motif of DAPK1. It is known, for example, that KLHL20 binds specifically to the activated pool of ULK1 to terminate autophagy (Liu et al., 2016).

KLHL20 has emerged as an interesting target for drug development with potential application in both oncology and Alzheimer's disease. Inhibition of KLHL20 would help to stabilize the tumor-suppressor proteins DAPK1 and PML (Lee et al., 2010, Yuan et al., 2011). It could also stabilize ULK1 to prolong autophagy, allowing greater clearance of potentially toxic misfolded proteins (Liu et al., 2016). The structure of KLHL20 at atomic resolution provides a robust template for structure-based drug design. Moreover, the identified DAPK1 peptide provides a valuable reagent for drug-screening assays based on peptide displacement. Of note, the hydrophobic interaction surface in KLHL20 includes an exposed cysteine residue (Cys356) that lies within 4 Å of the bound peptide (Figure 7D). This cysteine is accessible for modification and proximal to KLHL20 Trp326, another key DAPK1 interacting residue (Figures 5A and 7D). Thus, KLHL20 may be a promising target for screening against covalent inhibitor or fragment libraries.

**Figure 8:**
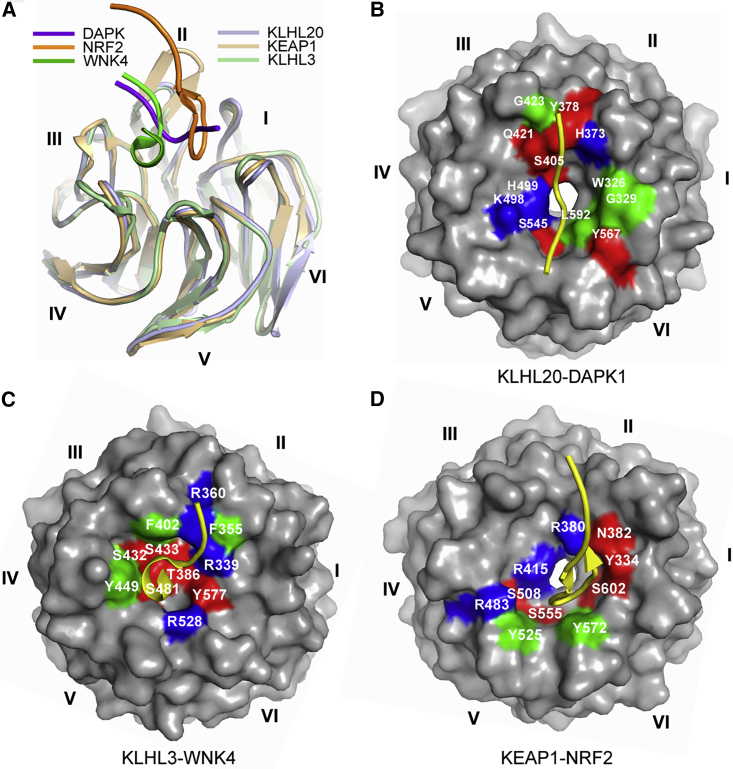
The Binding Mode of DAPK1 Is Distinct from Other Kelch-Substrate Complexes (A) Superposition of the complex structures of KLHL20-DAPK1 (light and dark purple), KEAP1-NRF2 (PDB: 2FLU, light and dark orange), and KLHL3-WNK4 (PDB: 4CH9, light and dark green). (B–D) Surface representations of the Kelch domains in the KLHL20-DAPK1 (B), KLHL3-WNK4 (C), and KEAP1-NRF2 (D) complexes with key contact residues highlighted by their binding characteristics (blue, basic residue; red, other polar; green, hydrophobic). The distinct surfaces and bound peptide conformations (yellow ribbons) highlight the rich variety of binding modes that can be established by the Kelch domain substrate pockets.

To date, few Kelch-substrate complexes have been structurally characterized, with the major examples being the KEAP1-NRF2 (Fukutomi et al., 2014, Lo et al., 2006, Padmanabhan et al., 2006) and KLHL3-WNK4 systems (Schumacher et al., 2014). The Kelch family proteins are relatively diverse in their primary sequences. Indeed, KLHL20 shares only 25%–50% sequence identity with other human Kelch domains. Superposition of the available complex structures shows that the substrate peptides are bound to their respective Kelch domains at different positions within the central pocket of the β propeller (Figure 8A). For example, the NRF2 peptide inserts toward KEAP1 blades I and VI, whereas the WNK4 peptide packs more toward KLHL3 blades III and IV (Figure 8A). These differences are supported by the variable BC loop lengths observed across the BTB-Kelch family (Canning et al., 2013). The patterning of hydrophobic and charged residues also differs across the different structures (Figures 8B–8D). The interaction surface in KLHL20 provides a notable hydrophobic contribution that should make it favorable for inhibitor development. Diverse substrate binding modes are also observed in the WD40 repeat class of E3 ligases as exemplified by the peptide co-structures of FBXW7 (Hao et al., 2007) and COP1 (Uljon et al., 2016), respectively.

For the most part, the study of protein-peptide complexes has focused on protein interaction domains with linear binding grooves. The Kelch domain β propeller offers a central pocket that can accommodate peptides with unexpected turns, twists, and helices. Overall, this feature is likely to increase the diversity of substrate interaction modes and allow for selective drug design. This is perhaps reflected in the prevalence of Kelch and WD40 domains within the family of Cullin-RING E3 ligases.

## STAR★Methods

### Key Resources Table

**Table:** 

REAGENT or RESOURCE	SOURCE	IDENTIFIER
**Antibodies**		

HRP conjugated Anti-His antibody	Merck Millipore	Cat#71840; RRID:AB_10947552
ANTI-FLAG® M2 Affinity Gel	Sigma-Aldrich	Cat#A2220; RRID:AB_10063035
Anti-Flag antibody, Sigma-Aldrich, F1804	Sigma-Aldrich	Cat#F1804; RRID:AB_262044
Anti-HA antibody	Biolegend	Cat#901501; RRID:AB_2565006
Anti-Myc antibody	Cell signalling	Cat#2040S; RRID:AB_2148465
anti-GAPDH antibody	Thermo Fisher	Cat#MA5-15738; RRID:AB_10977387

**Bacterial and Virus Strains**		

E.coli BL21(DE3)R3-pRARE2	SGC (Savitsky et al., 2010)	N/A

**Chemicals, Peptides, and Recombinant Proteins**		

DAPK01 Peptide ‘LLAMNLGLPDLVAKYNTSNGA’	Severn Biotech Ltd	N/A
DAPK02 Peptide ‘SKLRELGRRDAADFLLKA’	Severn Biotech Ltd	N/A
6xHis-KLHL20 Kelch/ untagged KLHL20 Kelch	This paper	N/A
KLHL3 Kelch	This paper	N/A
Crystallization peptide ‘LGLPDLVAKYN’	LifeTein	LT1342
SPOT peptide arrays	This paper	N/A
Ni Sepharose High Performance histidine-tagged protein purification resin	GE Healthcare	17526802
HiLoad® 16/600 Superdex® 200 pg	Sigma Aldrich	GE28-9893-35
Series S Sensor Chip CM5	GE Healthcare	BR100530
HBS-P buffer	GE Healthcare	BR100368

**Deposited Data**		

Co-structure of KLHL20-DAPK	https://rcsb.org	PDB: 6GY5
MyD88 structure used for homology modelling	(Lin et al., 2010)	PDB: 3MOP
KEAP1-NRF2 structure used for family comparisons	(Lo et al., 2006)	PDB: 2FLU
KLHL3-WNK4 structure used for family comparisons	(Schumacher et al., 2014)	PDB: 4CH9
KLHL12 structure used for phasing	(Canning et al., 2013)	PDB: 2VPJ

**Experimental Models: Cell Lines**		

HEK293T	ATCC	Cat#CRL-3216; RRID:CVCL_0063

**Oligonucleotides**		

KLHL20 Kelch fwd primer TACTTCCAATCCATGC AAGGACCAAGGACGAGACCACGG	Eurofin Genomics	N/A
KLHL20 Kelch rev primer TATCCACCTTTACTGTC ATTCACAATGTGTCATTTTAATAACTCCTACG	Eurofin Genomics	N/A
KLHL3 Kelch fwd primer TACTTCCAATCCATGAG CCTTCCCAAGGTCATGATTGTGG	Eurofin Genomics	N/A
KLHL3 Kelch rev primer TATCCACCTTTACTGTCA CAAGGACTTGTGAATCACGGC	Eurofin Genomics	N/A
KLHL20 FL (Myc) fwd primer GCATACGTCGACAT GGAAGGAAAGCCAATGCGC	Eurofin Genomics	N/A
KLHL20 FL (Myc) rev primer GCATACGTCGACTCA CAGATCCTCTTCTGAGATGAGTTTTTGTTCCCAAA TATGGGATTCACAATGTGT	Eurofin Genomics	N/A
DAPK01 FL (HA) fwd primer GCATACGTCGACATG ACCGTGTTCAGGCAGGAAAAC	Eurofin Genomics	N/A
DAPK01 FL (HA) rev primer CCGGCCGAATTCTCA AGCGTAATCTGGAACATCGTATGGGTACCGGGA TACAACAGAGCTAATGG	Eurofin Genomics	N/A
DAPK01 1338DL-AA_Mut_fwd primer CGCCATGAA CTTAGGCCTCCCTGCAGCAGTGGCAAAGTACAA CACCAGTAACGG	Eurofin Genomics	N/A
DAPK01 1338DL-AA_Mut_rev primer CCGTTACTG GTGTTGTACTTTGCCACTGCTGCAGGGAGGCCT AAGTTCATGGCG	Eurofin Genomics	N/A

**Recombinant DNA**		

pNIC28-Bsa4	SGC (Savitsky et al., 2010)	Addgene plasmid # 26103
pNIC28-Bsa4-KLHL3 Kelch domain	(Schumacher et al., 2014)	Addgene plasmid # 110251
pNIC28-Bsa4-KLHL20 Kelch domain	This paper	
pcDNA3.1(+)-DAPK1 full length	This paper	
pcDNA3.1(+)-DAPK1 mutant full length	This paper	
pcDNA3-N-Flag-LIC-KLHL20 Kelch domain	This paper	
pRK5-KLHL20 full length	This paper	

**Software and Algorithms**		

Phenix version 1.9	(Adams et al., 2010)	https://www.phenix-online.org/
CCP4	(Winn et al., 2011)	http://www.ccp4.ac.uk/download/index.php#os=windows
PyMOL	The PyMOL Molecular Graphics System, Version 2.0 Schrödinger, LLC	https://pymol.org/2/#download
PDBePISA	(Krissinel and Henrick, 2007)	http://www.ebi.ac.uk/pdbe/prot_int/pistart.html
GraphPad Prism version 7.02 for Windows	GraphPad Software, La Jolla California USA	www.graphpad.com
Molsoft ICM-Pro	Molsoft LLC.	http://www.molsoft.com/icm_pro.html

### Lead Contact and Materials Availability

Further information and requests for resources and reagents should be directed to and will be fulfilled by the Lead Contact, Alex Bullock (alex.bullock@sgc.ox.ac.uk).

### Experimental Model and Subject Details

#### Cell Lines

HEK293T cells (female) were cultured in high glucose Dulbecco’s Modified Eagle’s Medium (Sigma-Aldrich) with 5% Penicillin Streptomycin (ThermoFisher) and 10% Fetal Bovine Serum (Sigma-Aldrich) inside a 5% CO_2_ incubator at 37°C.

### Method Details

#### Constructs

For structural and biophysical studies, the Kelch domains of human KLHL20 (Uniprot Q9Y2M5 isoform 1, M303-E605) and KLHL3 (Uniprot Q9UH77 isoform 1, residues S298–L587) were cloned using ligation-independent cloning into the bacterial expression vector pNIC28-Bsa4 (GenBank accession number EF198106), which provides for an N-terminal hexahistidine tag and TEV cleavage site. For cellular assays, full length DAPK1 was cloned into pcDNA3.1(+) providing an N-terminal HA tag. The mutant DAPK1 (1336-‘LPDLV’ motif mutated to ‘LPAAV’) was created in the same vector by site directed mutagenesis. KLHL20 Kelch domain was cloned into pcDNA3-N-Flag-LIC providing an N-terminal Flag tag. Full length KLHL20 was cloned into pRK5 providing an C-terminal Myc tag. DNA sequences were verified by Source Bioscience Ltd.

#### Protein Expression and Purification

Plasmids were transformed into *E*. *coli* strain BL21(DE3)R3-pRARE2. Cells were cultured in LB broth at 37°C until OD_600_ reached 0.6. Recombinant protein expression was then induced by addition of 0.4 M isopropyl β-D-1-thiogalactopyranoside, followed by 18 hours continuous shaking at 18°C. Cells were harvested by centrifugation and lysed by sonication in binding buffer (50 mM HEPES pH 7.5, 500 mM NaCl, 5% glycerol, 5 mM imidazole) supplemented with 0.5 mM TCEP. Recombinant proteins were captured on nickel sepharose resin, washed with binding buffer and eluted by a stepwise gradient of 30-250 mM imidazole. Further clean-up was performed by size exclusion chromatography using a HiLoad 16/60 S200 Superdex column buffered in 50 mM HEPES pH 7.5, 300 mM NaCl, 0.5 mM TCEP. Finally, the eluted protein was purified by anion exchange chromatography using a 5 mL HiTrap Q column. Protein masses were confirmed by intact LC-MS mass spectrometry. Where required, the hexahistidine tag was cleaved overnight at 4°C using TEV protease.

#### Peptide Arrays (SPOT Assay)

Cellulose-bound peptide arrays were prepared employing standard Fmoc solid phase peptide synthesis using a MultiPep-RSi-Spotter (INTAVIS, Köln, Germany) as previously described (Picaud and Filippakopoulos, 2015). After array synthesis, membranes were incubated with 5% BSA to block free sites. The arrays were then incubated with 1 μM recombinant hexahistidine-tagged KLHL20 Kelch domain in PBS at 4°C overnight. Unbound protein was washed off in PBS buffer with 0.1% Tween 20 and bound protein was detected using HRP-conjugate anti-His antibody.

#### Surface Plasmon Resonance

Assays were performed at 25°C using a BIACORE S200 (GE Healthcare) surface plasmon resonance (SPR) instrument. The Kelch domains of KLHL20 and KLHL3 were immobilized on sensor chip CM5 (GE Healthcare) using amine coupling. Reference flow cells had no immobilized protein. Binding was monitored using a flow rate of 30 μL/min. The peptide analytes were prepared in HBS-P buffer (GE Healthcare). Data reported were after reference flow cell signal subtraction. Data were analyzed by one-site steady-state affinity analysis using the Biacore S200 Evaluation software and the fitting equation $Req=\frac{CRmax}{KD+C}+RI$. (RI, bulk refractive index contribution; *R*_max_, maximum response; *K*_D_, dissociation constant; C, analyte concentration). Peptides were obtained from Severn Biotech Ltd.

#### Protein Crystallization

The purified KLHL20 protein was concentrated to 12 mg/mL using a 10 kDa molecular-mass cut-off centrifugal concentrator in 50 mM HEPES pH 7.5, 300 mM NaCl and 5 mM TCEP buffer. The 11-residue DAPK1 peptide (LGLPDLVAKYN) was purchased from LifeTein and added in the same buffer to a final concentration of 3 mM. The protein-peptide mixture was incubated on ice for 1 hour prior to setting up sitting-drop vapour-diffusion crystallization plates. Micro-seed stocks were prepared from small KLHL20 crystals grown during previous rounds of crystal optimization. Those early crystals were transferred into an Eppendorf tube containing 50 μL reservoir solution and a seed bead (Hampton Research), then vortexed for 2 min. Seed stocks were diluted 500 fold before use. The best-diffracting crystals of the KLHL20 complex were obtained at 20°C by mixing 75 nL protein, 20 nL diluted seed stock and 75 nL of a reservoir solution containing 2 M sodium chloride and 0.1 M acetate buffer pH 4.5. Prior to vitrification in liquid nitrogen, crystals were cryoprotected by direct addition of reservoir solution supplemented with 25% ethylene glycol.

#### Structure Determination

Diffraction data for the KLHL20 Kelch domain-DAPK1 complex were collected on beamline I03 at Diamond Light Source, Didcot, U.K. Data were processed in PHENIX version1.9 (Adams et al., 2010). Molecular replacement was performed with PHENIX.Phaser-MR using KLHL12 (PDB 2VPJ chain A) as the search model. PHENIX.Autobuild was used to build the initial structural model. COOT (Emsley et al., 2010) was used for manual model building and refinement, whereas PHENIX.REFINE was used for automated refinement. TLS parameters were included at later stages of refinement. Tools in COOT, PHENIX and MolProbity (Chen et al., 2010) were used to validate the structures.

#### Homology Model

A homology model for the death domain of human DAPK1 was built in Molsoft ICM-Pro software using MyD88 (PDB 3MOP chain A, 25% sequence identity) as the structural template. The initial model was refined by energy minimization and side chain optimization in ICM-Pro (Molsoft) (Abagyan et al., 1997).

#### Co-immunoprecipitation of KLHL20 and DAPK1

HEK293T cells were cultured in high glucose Dulbecco’s Modified Eagle’s Medium (Sigma-Aldrich) with 5% Penicillin Streptomycin (ThermoFisher) and 10% Fetal Bovine Serum (Sigma-Aldrich) inside a 5% CO_2_ incubator at 37°C. KLHL20 Kelch domain (residues M303-T602) and full length DAPK1 constructs were transfected into HEK293T cells at 60% confluency with polyethylenimine. 40 hours after transfection, cells were harvested and lysed in the presence of protease and phosphatase inhibitors. Immunoprecipitation was performed using ANTI-FLAG® M2 Affinity Gel (Sigma-Aldrich). Results were analyzed using Western blotting (Anti-Flag antibody, Sigma-Aldrich, F1804; Anti-HA antibody, Biolegend, 901501; Anti-Myc antibody, Cell signalling, 2040S).

#### Cycloheximide Chase Assay

HEK293T cells were cultured as described above until 60% confluency. Full length KLHL20 and DAPK1 constructs were transfected with polyethylenimine. 24 hours after transfection, 100 μg/mL cycloheximide was added to inhibit protein synthesis. Cells were harvested at different time points – 0, 0.5h, 1h, 2h, 4h and 6h. Results were analyzed using Western blotting with corresponding antibodies. GAPDH level in each sample was also detected for control (anti-GAPDH antibody; Thermo Fisher, MA5-15738). Western blot band intensities were quantified using Image Studio Lite Ver 5.2 and normalized for the GADPH control.

### Quantification and Statistical Analysis

Diffraction data collection and structure refinement statistics are presented in Table 1.

### Data and Code Availability

The crystal structure coordinates have been deposited in the Protein Data Bank (PDB) under ID code 6GY5.

## Supplemental Information

Document S1. Figures S1–S3

Document S2. Article plus Supplemental Information
