# Supplementary material for: Structural Basis for Recruitment of DAPK1 to the KLHL20 E3 Ligase
Source: Structure. 2019 Sep 3;27(9):1395–1404.e4. doi: 10.1016/j.str.2019.06.005 (PMC6720452; doi:10.1016/j.str.2019.06.005)
Supplement: Document S1. Figures S1–S3 [file mmc1.pdf]

Structure, Volume *27*

Supplemental Information

Structural Basis for Recruitment of DAPK1 to the KLHL20 E3 Ligase

Zhuoyao Chen, Sarah Picaud, Panagis Filippakopoulos, Vincenzo D'Angiolella, and Alex N. Bullock

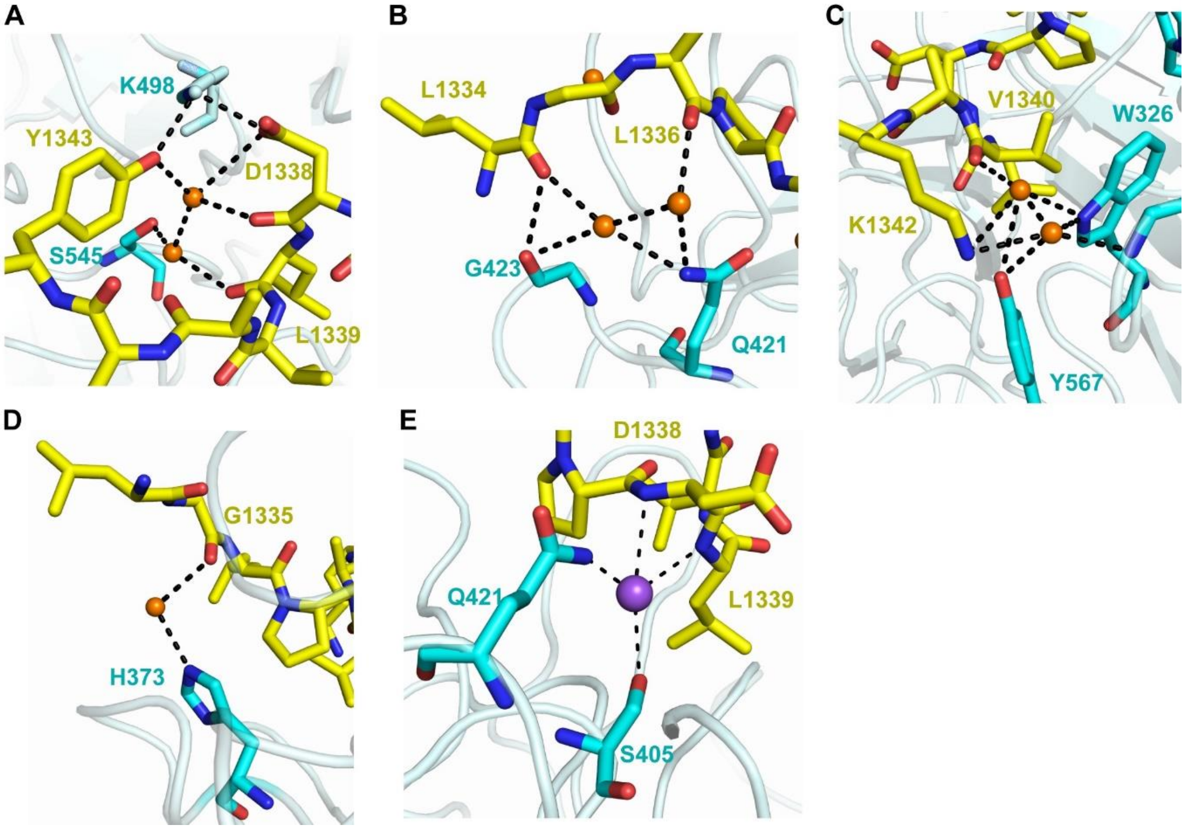

Figure S1. Indirect contacts between KLHL20 and DAPK1, related to Figure 4. (A-D) Water-bridged hydrogen bonds in the protein-peptide interface. Waters are shown as orange spheres. **(E)** A sodium ion (purple sphere) is proximal for electrostatic interactions with the side chains of KLHL20 Ser405 and Gln421 as well as the backbone atoms of DAPK1 D1338 and L1339.

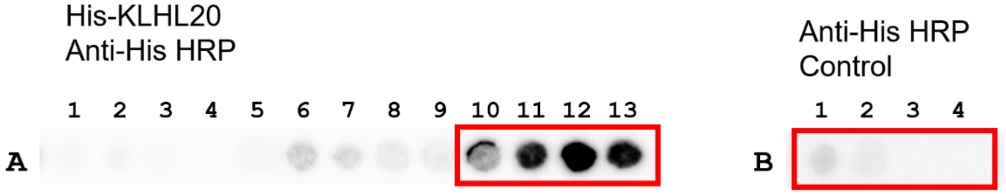

SPOT	Peptide	Residues
A1	V-G-D-Q-R-T-E-F-L-G-A-A-P-L-G	654-668
A2	Q-R-T-E-F-L-G-A-A-P-L-G-P-P-V	657-671
A3	E-F-L-G-A-A-P-L-G-P-P-V-S-P-P	660-674
A4	G-A-A-P-L-G-P-P-V-S-P-P-H-V-S	663-677
A5	P-L-G-P-P-V-S-P-P-H-V-S-T-F-K	666-680
A6	P-P-V-S-P-P-H-V-S-T-F-K-T-R-S	669-683
A7	S-P-P-H-V-S-T-F-K-T-R-S-A-K-G	672-686
A8	H-V-S-T-F-K-T-R-S-A-K-G-F-G-A	675-689
A9	T-F-K-T-R-S-A-K-G-F-G-A-R-G-P	678-692
A10	T-R-S-A-K-G-F-G-A-R-G-P-D-V-L	681-695
A11	A-K-G-F-G-A-R-G-P-D-V-L-S-P-A	684-698
A12	F-G-A-R-G-P-D-V-L-S-P-A-M-V-A	687-701
A13	R-G-P-D-V-L-S-P-A-M-V-A-L-S-N	690-704
B1	T-R-S-A-K-G-F-G-A-R-G-P-D-V-L	681-695
B2	A-K-G-F-G-A-R-G-P-D-V-L-S-P-A	684-698
B3	F-G-A-R-G-P-D-V-L-S-P-A-M-V-A	687-701
B4	R-G-P-D-V-L-S-P-A-M-V-A-L-S-N	690-704

Figure S2. SPOTs peptide array for EPAS1, related to Figure 1. Each spot was printed as a 15-mer EPAS1 peptide with a 3 residue frameshift at each consecutive position. SPOTs array A1-13 was incubated with purified 6xHis-KLHL20 Kelch domain, washed and then KLHL20 binding detected using anti-His HRP-conjugated antibody. Binding was observed at peptides containing a ‘GPDVL’ motif. As a control, duplicate SPOTs array B1-B4 was probed with antibody alone.

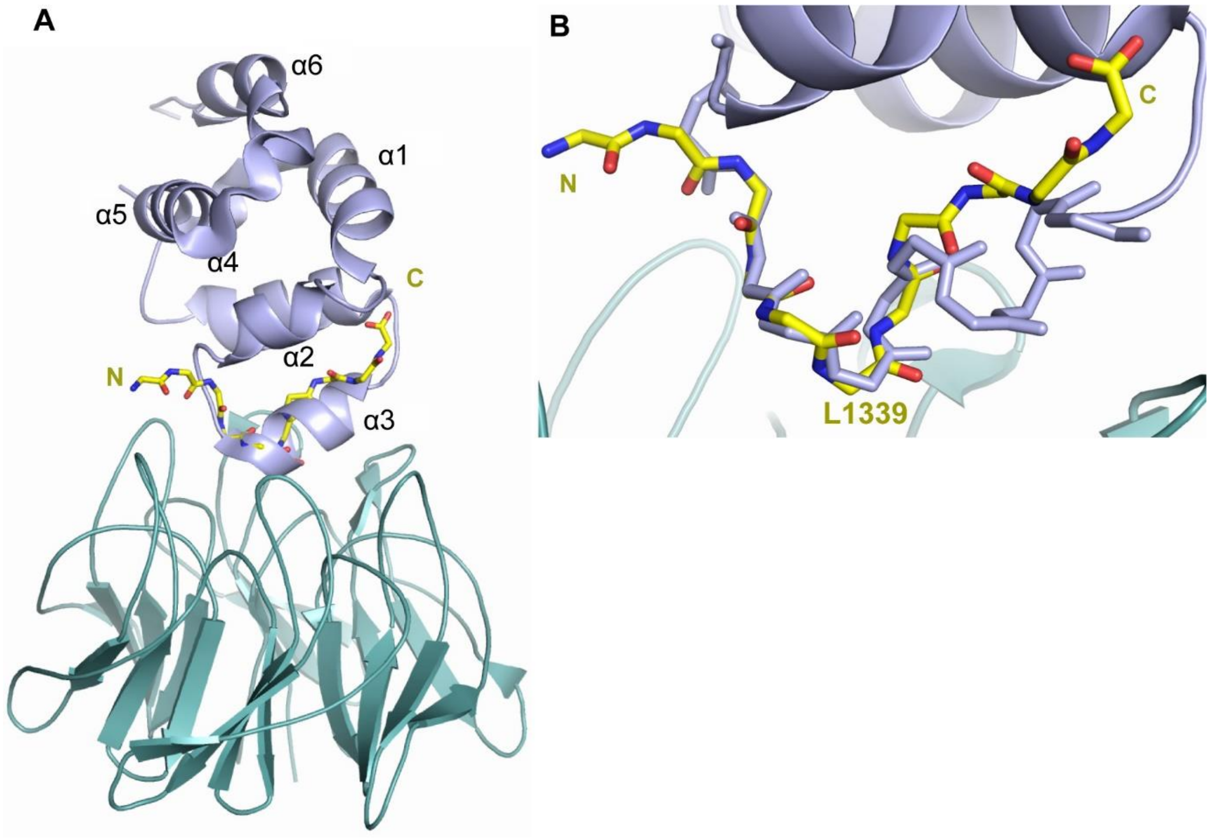

Figure S3. Structural comparison between the DAPK1 peptide and MyD88 death domain, related to Figure 7. (A) Superposition of the KLHL20 Kelch domain-DAPK1 structure (cyan/yellow) and the structure of the MyD88 death domain (light purple; PDB 3MOP chain A) based on the DAPK1 'LPDLV' motif. (**B**) Close-up view showing good agreement between the helical conformation of the 'LPDLV' motif in the crystallized DAPK1 peptide and the MyD88 structure.
